# New Polymer Inclusion Membrane in the Separation of Nonferrous Metal Ion from Aqueous Solutions

**DOI:** 10.3390/membranes10120385

**Published:** 2020-11-30

**Authors:** Ilona Pyszka, Elzbieta Radzyminska-Lenarcik

**Affiliations:** Faculty of Chemical Technology and Engineering, UTP University of Science and Technology, Seminaryjna 3, 85-326 Bydgoszcz, Poland; ilona.pyszka@utp.edu.pl

**Keywords:** acetylacetone derivatives, extraction, heavy metals, polymer inclusion membrane

## Abstract

The new polymer inclusion membrane (PIM) with ethylenediamine-bis-acetylacetone (EDAB-acac) matrix was used for the separation of Zn(II) solutions containing non-ferrous metal ions (Co(II), Ni(II) Cu(II), Cd(II)). The effective conditions for carrying out transport studies by PIMs were determined on the basis of solvent extraction studies. The values of the stability constants and partition coefficients of M(II)-EDAB-acac complexes were determined from the extraction studies. The stability constants increase in series Ni(II) < Cu(II) < Co(II) < Cd(II) < Zn(II), and their logarithms are 8.85, 10.61, 12.73, 14.50, and 16.84, respectively. The transport selectivity of the PIMs were: Zn(II) > Cd(II) > Co(II) > Cu(II) > Ni(II). The established stability constants of the complexes also decrease in this order. The values of three parameters: initial flux, selectivity coefficient, and recovery factor of a given metal after 12 h were selected for the comparative analysis of the transport process. The highest values of the initial fluxes were received for Zn(II), Cd(II), and Co(II). They are, depending on the composition of the mixture, in the range 9.87–10.53 µmol/m^2^, 5.26–5.61 µmol/m^2^, and 7.43–7.84 µmol/m^2^ for Zn(II), Co(II), and Cd(II), respectively. The highest recovery factors were observed for Zn(II) ions (90–98.0%). For Cd, Co and Cu, the recovery factors are high and are within the range 76–83%, 64–79%, and 51–66%, respectively.

## 1. Introduction

Heavy metals like Zn, Cu, Pb, Ni, Cd, Hg, Cr, etc., due to their toxicity, cause various environmental problems. Industrial wastewater containing heavy metals is produced by almost all industries [1,2]. These include electroplating, electrolysis, conversion coating, anodizing-cleaning, machining, and etching. All these processes produce large amounts of wastewater, sludge, and other waste that can be classified as hazardous and require treatment [3,4].

The conventional processes for removing heavy metals from wastewater include, e.g., chemical precipitation, flotation, adsorption, ion exchange, and solvent extraction [5,6,7,8,9,10,11,12,13]. The elimination of heavy metal ions from waste is carried out not only for environmental protection but also to recover economically valuable metals [11,12,13,14,15,16,17]. Zinc, as well as copper, nickel, chromium, and cadmium are considered very important metals for the development of the economy [18]. Their use is steadily increasing, and the use of ores for their production is limited. Therefore, they inevitably must be recovered from waste materials. All forms of waste products, including wastewater, slimes, and tailings, can be considered as raw materials for metal recovery. Thus, developing separation techniques for determination and removal of these metals from aqueous solutions becomes an urgent necessity. In recent years, membrane separation techniques have been increasingly used to remove heavy metals [19,20,21], toxic metals [22,23,24] non-ferrous metals [25,26,27], and also for metal plating wastewater treatment [14,15,28,29] and other liquid effluents [30].

Commercial metal ion carriers most often used in membrane techniques enable effective separation of these ions, but their selectivity is low. Therefore, new coordinating compounds, which will allow the selective release of metal ions from aqueous solutions, are constantly being searched for. [31,32]. 

In recent years, β-diketone derivatives (acetylacetone derivatives, acac) has been effectively used as both extractants and metal ion carriers [33,34,35,36,37]. An example of this might be the wide use of LIX^®^ reagents. LIX^®^ reagents are known to be very selective towards Cu(II) ions [36,37]. The transport properties for Cu(II) of polymer inclusion membrane (PIMs) doped with commercially available LIX^®^ reagents (LIX^®^ 84-I, LIX^®^ 984, LIX^®^ 54-100) were studied by de San Miguel [36]. Transport efficiency of Cu(II) ions by PIMs doped with LIX^®^ in sulfate solutions decreased as follows: LIX^®^ 984 > LIX^®^ 84-I > LIX^®^ 54-100 [36]. LIX^®^ 54-100 contains 6 isomeric 1-phenyldecane-1,3-dione with various structures of the alkyl group, and additionally heptane-8,10-dione, 1,3-diphenylpropane-1,3-dione, and unknown compounds with a carbonyl group [38].

For the separation of a mixture of Zn(II), Cd(II), and Ni(II) ions as a carrier in liquid membranes (LMs), β-diketone derivatives such as LIX-54, (p-hexylphenyl)-1,3-butanedione, and 1-phenyl-2-hexyl-1,3-butanedione were used. [39].

3-substituted derivatives of acac were used as extractant of Cu(II) ions [40]. Zn (II) ions were effectively separated from the Zn-Cu-Co-Ni mixture in the transport process by PIMs doped with both 3-propyl-acac and 3-benzyl-acac [41]. 

The selectivity of the transport process of Cu(II) ions from a mixture of Co(II), Ni(II), and Cu(II) ions across PIMs with aromatic β–diketones (benzoyl-acac and dibenzoylmethane) as carriers has been also investigated [42].

The aim of the work was to investigate the usefulness of EDAB-acac in the separation of Zn(II) from a mixture of non-ferrous metal ions such as Co(II), Ni(II) Cu(II), and Cd(II), and evaluation of the suitability of EDAB-acac for the separation of zinc from a mixture. To achieve this aim, the solvent extraction (SX) as well as transport of non-ferrous ions across PIMs with ethylenediamine-bis-acetylacetone (EDAB-acac) were investigated. Extraction studies will allow us to determine the optimal conditions for transport by PIMs (pH of the feeding phase, partition constant, and stability constant of the complexes). Extraction studies will allow us to determine the optimal transport conditions through PIMs (pH of the feeding phase, partition constant “s,” and complex stability constants). Another aim was to calculate the values of the parameters characterizing the transport of Zn(II), Co(II), Ni(II) Cu(II), and Cd(II) ions in their 5-, 4- and 3-component mixtures in order to determine the Zn(II) separation coefficient in relation to the remaining ions, as well as to determine the recovery percentage of individual metals. 

Previously, EDAB-acac was used as a carrier in PIMs to separate Zn from a Zn-Cr-Ni mixture [43]. 

## 2. Experimental

### 2.1. Reagents and Equipment

Inorganic chemicals: potassium, zinc(II), nickel(II), copper(II), cadmium(II), and cobalt(II) nitrates, nitric acid (HNO_3_) were of analytical grade and were purchased from POCh (Gliwice, Poland). Ammonia buffer was prepared from ammonia (NH_3_) and ammonium nitrate (NH_4_NO_3_) (both analytical reagent grade, POCh, Gliwice, Poland). Aqueous solutions were prepared with double-distilled water (conductivity 0.1 µS/m). Concentration of the heavy metal ions was determined by titration with EDTA (POCh, Gliwice, Poland). The potassium nitrate concentration was determined gravimetrically as sulfate. 

**Figure 1:**
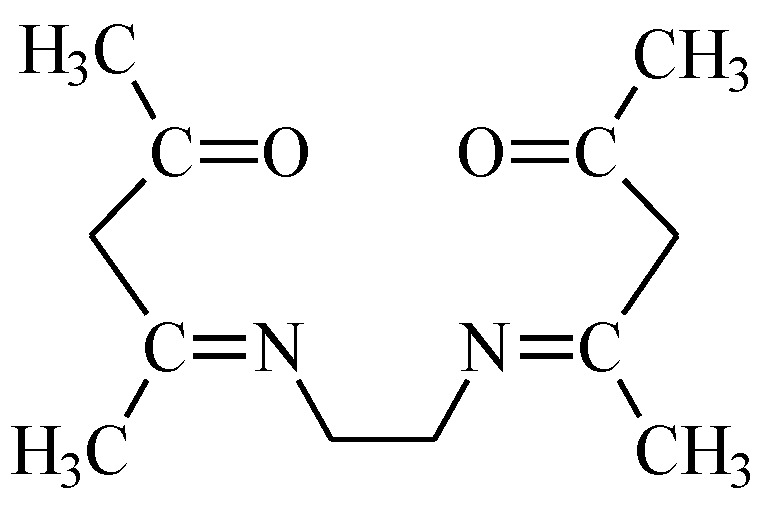
Ethylenodiamino-bis-acetylacetone (EDAB-acac) formula.

Ethylenodiamino-bis-acetylacetone (EDAB-acac) (Figure 1) was synthesized according to the procedure described in the paper [44]. Its characteristics are given in the paper [43]. 

Cellulose triacetate (CTA), *o*-nitrophenyl pentyl ether (*o-*NPPE), and dichloromethane (Fluka, Busch, Switzerland) were used without further purification.

The pH-meter (PHM 250 (Radiometer, Copenhagen, Denmark) equipped with a glass-calomel combination electrode C 2401-8 (Radiometer, Copenhagen, Denmark) was calibrated using commercial buffer solutions (Radiometer, Copenhagen, Denmark) having a pH of 7.00 ± 0.01 and 9.21 ± 0.01. 

Metal ions concentrations in aqueous phases were analyzed with AAS 240FS Spectrometer, Agilent, Santa Clara, CA, USA (AAS—atomic absorption spectroscopy).

A Varian, Cary 50 spectrophotometer (LabMakelaar Benelux B.V., Zevenhuizen, The Netherlands) was used for recording the absorption spectra of the Cu(II) and Co(II) complexes in the aqueous and organic phase over the visible region.

### 2.2. Procedure for Determination of Dissociation Constants (pK_a_)

The dissociation constants (*pK_a_*) of EDAB-acac was determined by potentiometric titration of the acid solution with standard solution of 0.005 mol/dm^3^ HNO_3_ at 25 °C ± 0.5 °C. Measurements were taken for two EDBA-acac concentrations (0.06 mol/dm^3^ and 0.05 mol/dm^3^) in the acid solution. Three series of measurements were run.

### 2.3. Liquid-Liquid Extraction Procedure (SX)

The measurements were carried out at 20 °C and at a fixed ionic strength (0.5 mol/dm^3^) maintained in the aqueous phase with KNO_3_. Whereas constant pH was maintained with ammonia buffer (NH_3_ + NH_4_NO_3_, 1:1). Before extraction, the concentrations of Co(II), Ni(II), Cu(II), Zn(II), and Cd(II) ions in the aqueous phase were kept constant (0.01 mol/dm^3^) and the ligand (EDBA-acac) concentration in the organic phase (methylene chloride) was varied (from 0.01 to 0.03 mol/dm^3^). Equal volumes of organic and aqueous phases (phase volume ratio O/A = 1) were mechanically shaken for 20 min. After establishing equilibrium, the phases were separated. Equilibrium pH of aqueous phases were measured. In the aqueous phase, metal concentrations were measured. UV-VIS spectra of both aqueous and organic phases were recorded.

### 2.4. Polymer Inclusion Membrane

PIMs were prepared as reported in the earlier paper [43]. During further testing [43], the membrane contained 2.7 cm^3^ o-NPPE/1g CTA and 0.8 mol/dm^3^ of EDAB-acac based on plasticizer. 

### 2.5. Transport Studies

Transport experiments were carried out in the system described in earlier papers [41,42,43,45,46] at 20 ± 0.2 °C. The feed phase was an aqueous solution of metal salts (*C*_0*,M*_ = 0.001 mol/dm^3^ each) with pH = 7.8 maintained by ammonia buffer and controlled by pH-meter (pH-meter PHM 250, Radiometer, Copenhagen, Denmark) with a combination pH electrode (C 2401-8 Radiometer, Copenhagen, Denmark). The receiving phase was deionized water, pH = 6.8. At the feed and receiving phases, metal ions concentrations were measured.

## 3. Results and Discussion

### 3.1. Determination of Dissociation Constants (pK_a_)

EDAB-acac dissociate according to the reaction:
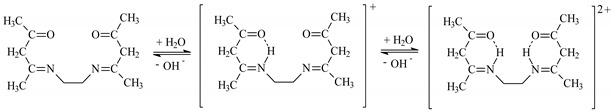
EDAB-acac + H_2_O ↔ HEDAB-acac^+^ + OH^−^ HEDAB-acac^+^ + H_2_O ↔ H_2_EDAB-acac^2+^ + OH^−^(1)

The equilibrium constant (*pK_a_* of EDAB-acac) is defined:

(2) $$\begin{array}{c} K_{a1}=\frac{\left[{\mathrm{HEDAB}-\mathrm{acac}}^{+}\right]\left[OH^{-}\right]}{\left[EDAB-\mathrm{acac}\right]}\\ K_{a2}=\frac{\left[{\mathrm{HEDAB}-\mathrm{acac}}^{2+}\right]\left[OH^{-}\right]}{\left[{\mathrm{HEDAB}-\mathrm{acac}}^{+}\right]} \end{array}$$

The *pK_a_* values were determined by potentiometric method described by Braibanti [47] and summarized in Table 1 together with *pK_a_* values for other acac derivatives.

Higher values of *pK_a_* provide weaker acid properties. The strongest alkali (Table 1) is EDAB-acac which is due to the presence of two amine groups in the molecule.

### 3.2. Solvent Extraction of Metal Ions by EDAB-acac

In order to understand the transport of metal ions across the PIMs doped with EDAB-acac as an ion carrier better, it was necessary to perform solvent extraction studies with EDAB-acac as extractant. Solvent extraction of individual metal ion (Co(II), Ni(II), Cu(II), Zn(II), Cd(II)) solutions from ammonia solutions was carried out. 

The distribution ratio (*D_M_*) of metal ion is defined as:

(3) $$D_{M}=\frac{C_{M\left(org\right)}}{C_{M\left(aq\right)}}=\frac{C_{0,M}-C_{M}}{C_{M}}$$

where: *C*_0*,M*_ and *C_M_* denote analytical metal ion concentrations in the aqueous phase before and after the extraction equilibrium was achieved, respectively. For each case, *D_M_* was calculated from the Formula (3).

**Figure 2:**
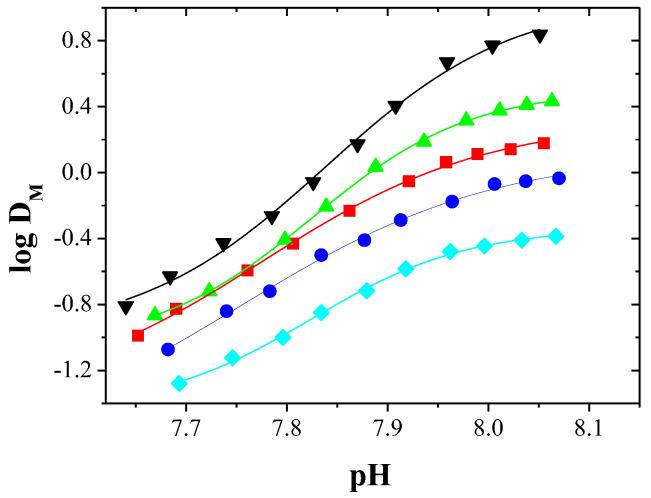
Logarithm of distribution ratio of ▼—Zn(II), ▲—Cd(II), ■—Co(II), ●—Cu(II), and ♦—Ni(II) complexes with EDAB-acac vs. equilibrium pH of the aqueous phase.

Figure 2 shows the effect of pH on the logarithm of distribution ratio (*D_M_*) of metal ions between the aqueous and organic phases (methylene chloride) for solutions containing individual metal ions.

Figure 2 shows that the distribution ratio of each metal ion complex increases with increasing pH of the aqueous phase. According to the *D_M_* values, the extraction efficiency depends on the type of metal ion and increase in order Ni(II) < Cu(II) < Co(II) < Cd(II) < Zn(II).

The pH_1/2_ values corresponding to 50% metal extraction for Zn, Cd, and Co are equal 7.84, 7.88, and 7.94, respectively.

The percentage extraction of each metal ion calculated using formula:

(4) $$\%E=\frac{D_{M}\cdot 100\%}{D_{M}+V_{aq}/V_{org}}$$

where: *V_aq_* and *V_org_*–volumes of aqueous and organic phases.

The highest percentage extraction of zinc (87%), cadmium (73%), cobalt (60%), copper (48%), and nickel (29%) obtained for pH c.a. 8.0.

**Figure 3:**
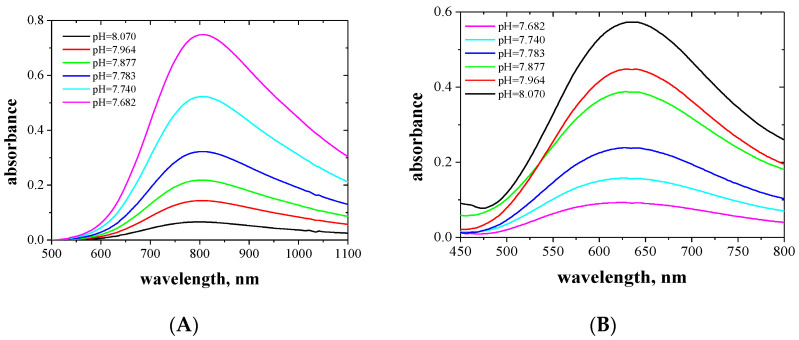
Absorption spectra of aqueous (**A**) and organic (**B**) phases after extraction of the Cu(II) complexes with EDAB-acac in methylene chloride together with the corresponding pH of the aqueous phase.

**Figure 4:**
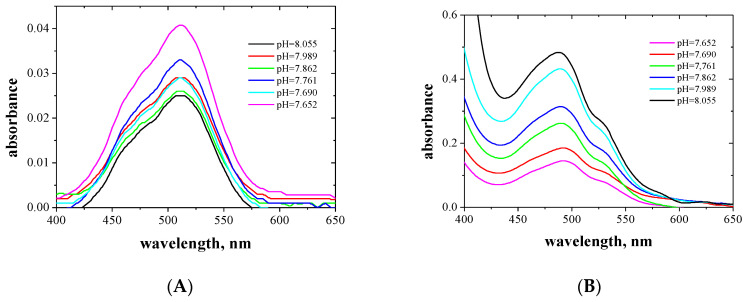
Absorption spectra of aqueous (**A**) and organic (**B**) phases after extraction of the Co(II) complexes with EDAB-acac in methylene chloride together with the corresponding pH of the aqueous phase.

The absorption spectra of the aqueous and organic phases after extraction Cu(II) and Co(II) complexes with EDAB-acac were measured in the wavelengths range of 400 to 850 nm, and are presented in Figure 3 and Figure 4, respectively.

The Cu(II) complexes with EDAB-acac are blue and deep-blue in the aqueous and organic phases, respectively. In the organic phase, the maximum absorption increases with increasing ligand concentration. The absorption maxima for the aqueous and organic phases occur at wavelengths of 805 (Figure 3A) and 635 nm (Figure 3B), respectively. One maximum absorption testifies to the formation of a single complex with octahedral symmetry. A similar phenomenon was observed in the works [49,50,51,52].

The Co(II) complexes with EDAB-acac are pink and orange in the aqueous and organic phases, respectively. For the aqueous phase, the maximum absorption occurs at a wavelength of 509 nm. In the organic phase, the maximum absorption occurs at a wavelength of 489 nm, however, as the ligand concentration increases, a second peak appears at a wavelength of 530 nm. In the case of Co (II), there are two maximum absorptions, which prove the formation of two types of complexes. Maximum at a wavelength of approx. 480 corresponds to octahedral complexes, while the band at higher wavelengths approx. 530 corresponds to tetrahedral complexes. Changes in the symmetry of the coordination sphere have been observed in the case of Co (II) complexes with alikoimidazoles [53] and are also described in [51] and also e.g., in [54,55].

### 3.3. Determination of the Stability Constants

On the basis of SX studies, the stability constants of investigated M(II) with EDAB-acac complexes formed in solution were determined by liquid-liquid partition method. This method was previously used to determine the stability and partition constants of Cu(II) [49,56], Co(II) [53,57], Ni [58], Zn [59,60], Cd [61] with alkyl imidazoles, and Cu(II) with alkyl derivatives of pentane-2-dione (acac) [40].

The values of the stability constants of the investigated complexes were calculated on the basis of the modified Rydberg formula:

(5) $$D_{M}=\frac{P_{n}\beta_{n}{\left[L\right]}^{n}+P_{n+1}\beta_{n+1}{\left[L\right]}^{n+1}+\ldots ..+P_{N}\beta_{N}{\left[L\right]}^{N}}{\sum_{n=0}^{n=N}\beta_{n}{\left[L\right]}^{n}}$$

where: *P_n_, β_n_* and [*L*] denote the partition constant, stability constant, and the free ligand concentration (mol/L) in the aqueous phase, respectively, and n is the number of ligand particles in the first metal ion complex which is hydrophobic to the extent that it is possible for it to pass freely into the organic phase [62,63].

Stability constants, *β_n_*, the M(II) -EDAB-acac complexes determined on the basis of the Equation (5) are collected in Table 2 together with the stability constants, previously determined for Cu(II), Co(II), Ni(II), Zn(II) and Cd(II) complexes with pentane-2-dione (acac) [40,48], and Cu(II) with 3-substituted acetylacetone [40].

The stability of M(II)-EDAB-acac complexes is the highest, compared to the complexes shown in Table 2 of the ligands. The increase in stability may be caused by the higher alkalinity of the EDAB-acac molecule. Both acetylacetone and its derivatives can form complexes with the metal ions with the coordination number (CN) 4 having a square planar geometry. As a result, very stable 5-membered chelate rings are formed Equation (6).



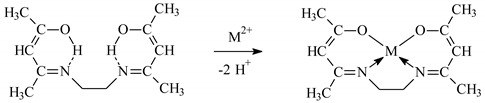
(6)


The stability of the complex also depends on the properties of the central ion. For complexes with EDAB-acac, it decreases respectively: Zn(II) > Cd(II) > Co(II) > Cu(II) >Ni(II).

In the case of Zn (II), Co (II), and Cd (II) ions, an additional phenomenon is the ease of changing CN from 6 to 4, and thus the geometry of the coordination sphere from octahedron to tetrahedron or square planar [43,53,57,59,60,61]. This is illustrated by Equation (7):

for Zn(II), Cd(II), Co(II):  [M(H_2_O)_6_]^2+^ + n L ↔ [M(H_2_O)_4-n_L_n_]^2+^ + (n + 2) H_2_O (7)

Although Cu(II) ions have a plastic coordination sphere [64], they rarely form complexes with planar structure [56]. The Ni(II) ions form 6-coordination complexes, because they have a rigid octahedral structure which is hard to deform.

The formation of tetrahedral or square planar complexes enhances the extraction of Zn(II), Cd(II) and Co(II) [12,15,53,57,59,60,61].

### 3.4. Transport across PIMs

For the study of the transport of non-ferrous metal ions by PIMs with EDAB-acac as a carrier, membranes composed of: 2.7 cm^3^ o-NPPE/1g CTA, and 0.8 mol/dm^3^ of EDAB-acac based on plasticizer were examined. This membrane composition was chosen as optimal for the separation of Zn from the Zn-Cr-Ni mixture [43].

The kinetics of metal ions transport across membrane was described by the Equation (8):

(8) $$ln\;\frac{C_{0,M}^{\;\;}-C_{M}}{C_{M}}=\;-kt$$

where: *C_M_* is the metal ions concentration (mol/dm^3^) in the feed phase at a given time, *C*_0*,M*_ is the initial metal ions concentration, *k* is the rate constants (s^−1^), and *t* is the time of transport (s) [65].

**Figure 5:**
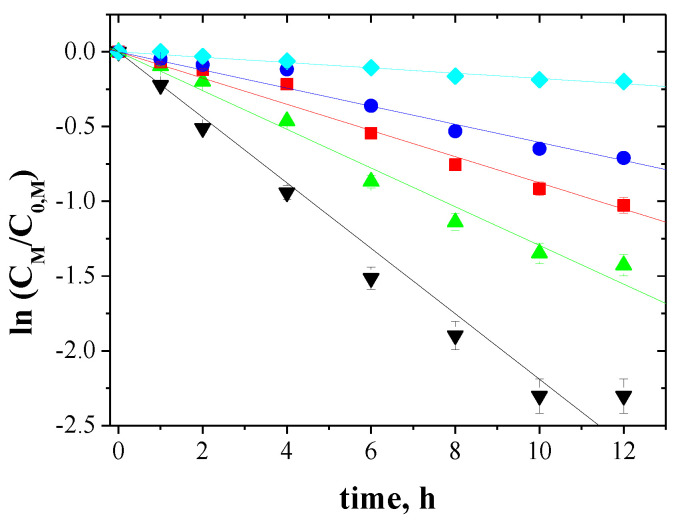
Kinetics of the transport across PIMs containing EDAB-acac for 5-component mixture of ▼-Zn(II), ▲-Cd(II), ■-Co(II), ●-Cu(II), and ♦-Ni(II).

**Figure 6:**
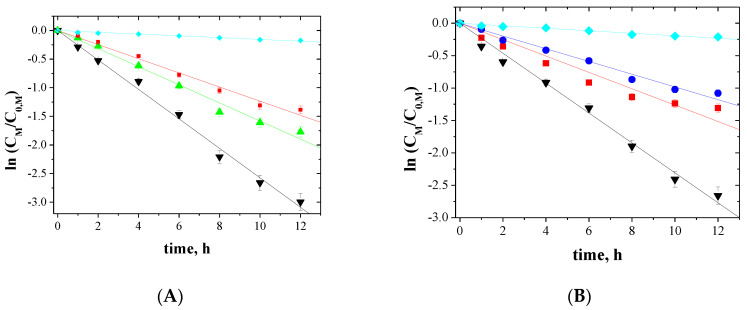
Kinetics of the transport across PIMs containing EDAB-acac for 4-component mixture of ▼-Zn(II), ▲-Cd(II), ■-Co(II), and ♦-Ni(II) (**A**); ▼-Zn(II), ■-Co(II), ●-Cu(II), and ♦-Ni(II) (**B**).

**Figure 7:**
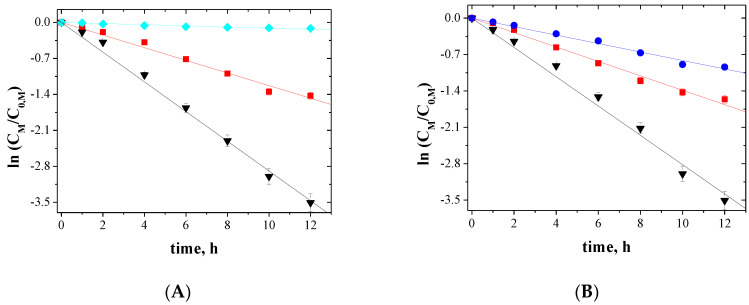

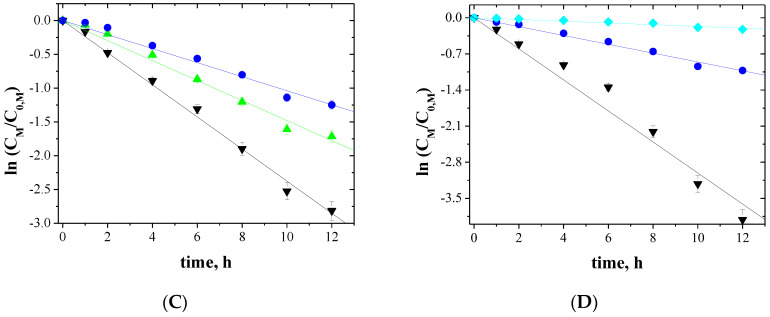
Kinetics of the transport across PIMs containing EDAB-acac for 3-component mixture of ▼-Zn(II), ■-Co(II), and ♦-Ni(II) (**A**); ▼-Zn(II), ■-Co(II), and ●-Cu(II) (**B**); ▼-Zn(II), ▲-Cd(II), and ●-Cu(II) (**C**); ▼-Zn(II), ●-Cu(II), and ♦-Ni(II) (**D**).

The relationship between ln(*C_M_/C*_0*,M*_) and the time of metal ions transport across PIMs for 5-, 4- and 3-component mixtures is shown in Figure 5, Figure 6 and Figure 7, respectively.

The correlation between ln(*C_M_/C*_0*,M*_) and time was linear, which was confirmed by the high correlation coefficient (R2) ranging from 0.9733 to 0.9985. Thus, the kinetics of transport is a first-order reaction to the concentration of metal ions. For the blank experiment, no transport was detected for more than 12 h of the continuous process run.

### 3.5. Initial Fluxes, Order and Separation Coefficients for Non-Ferrous Metal Transport across PIMs

A linear dependence of ln(*C_M_/C*_0*,M*_) in the feed phase versus time was obtained and the permeability coefficient was calculated from the slope of the straight line that fits the experimental data. The initial flux (*J_0_*) was determined as being equal to:

(9) $$J_{0}=\;P\cdot c_{0}\;$$

The selectivity coefficient (*S_M_*_1*/M*2_) was defined as the ratio of initial fluxes for *M*1 and *M*2 metal ions, respectively:

(10) $$S_{M_{1}/M_{2}\;}=J_{0,M_{1}}/J_{0,M_{2}}$$

Initial fluxes and selectivity coefficients for competitive transport of non-ferrous metal ions across PIMs doped with EDAB-acac are summarized in Table 3.

As shown by the results displayed in Table 3, Zn(II) ions transport is the highest. For a multi-component mixture, the initial fluxes of metal ions transported across PIMs doped with EDAB-acac decrease in the following order: Zn(II) > Cd(II) > Co(II) > Cu(II) > Ni(II).

In the case of Zn(II), Cd(II) and Co(II), the higher values of the initial fluxes are perhaps due to the ease of changing the symmetry of the coordination sphere from an octahedron to a square planar as well as with the higher stability constants of their complexes with EDAB-acac.

Selectivity coefficients (S) Zn(II)/Cd(II) and Zn(II)/Co(II) decrease with the increasing amount of ions in the mixture. They are about 1.3 for Zn/Cd and they range 1.7–2.0 for Zn/Co.

The Zn/Cu selectivity coefficient increases with the increase of the number of components in the mixture from 1.6 for Zn-Cu-Ni mixture and 2.5 for Zn-Co-Cu mixture to 3.2 for 5-component (Zn-Cd-Co-Cu-Ni) mixture. High separation coefficients were obtained for Zn/Ni. Those are 21, c.a. 25 and 46 for a 3-, 4-and 5-components mixture, respectively.

### 3.6. Recovery of Metal

In order to describe the efficiency of metal removal from the feed phase, the recovery coefficient (*RF*) was calculated from Equation (11):

(11) $$RF=\;\frac{C_{0,M}-C}{C_{0,M}}\cdot \;100\%$$

where *C* is the metal ions concentration (mol/dm^3^) in the feed phase after 12 h transport.

Table 4 shows the values of the recovery coefficient Zn(II), Cd(II), Co(II), Cu(II), and Ni(II) ions from the feed phase during the 12-h transport across PIMs with EDAB-acac.

Recovery factor of each metal ion depends on the composition of the mixture. The lowest recovery factors for each metal ion were obtained for the 5-component mixture.

In each case, recovery factor of Zn(II) ions was the highest, amounting to 90–98%.

For Cd, Co and Cu, the recovery factors are high and are within the range 76–83%, 64–79%, and 51–66%, respectively.

Ni(II) ions are transported to a small extent across PIMs with EDAB-acac. They practically remain in the feed phase.

Previously, PIMs doped with EDAB-acac were used to separate Zn(II) from Zn-Cr-Ni mixture [43]. The selectivity coefficients equal to 1.2 and 15.9 was determined for Zn(II)/Cr(III) and Zn(II)/Ni(II), respectively. After 24 h transport the recovery factor of Zn(II), Cr(III), and Ni(II) were 90, 65, and 6%, respectively [43].

## 4. Conclusions

Ethylenodiamino-bis-acetylacetone forms very stable complexes with Zn(II), Cu(II), Co(II), Ni(II) and Cd(II). The stability constants of these complexes increase in order: Ni(II) > Cu(II) > Co(II) > Cd(II) > Zn(II). For individual solution of metal ions the highest percentage extraction of zinc (87%), cadmium (73%), cobalt (60%), copper (48%), and nickel (29%) obtained for pH c.a. 8.0.

The new CTA membrane with ethylenodiamino-bis-acetylacetone may be useful for the separation of zinc from non-ferrous metal solutions, such as Cu(II), Co(II), Ni(II), and Cd(II).

The initial fluxes of metal ions transport across PIMs with EDAB-acac decrease in the following order: Zn(II) > Cd(II) > Co(II) > Cu(II) > Ni(II). Selectivity coefficients Zn(II)/Cd(II) and Zn(II)/Co(II) decrease with an increasing amount of ions in the mixture. They are about 1.3 for Zn/Cd and they are in the range of 1.7–2.0 for Zn/Co. The Zn/Cu selectivity coefficient increases with the increase of the number of components in the mixture from 1.6 for Zn-Cu-Ni mixture and 2.5 for Zn-Co-Cu mixture to 3.2 for 5-component (Zn-Cd-Co-Cu-Ni) mixture. High separation coefficients were obtained for Zn/Ni. Those are 21, c.a. 25 and 46 for a 3-, 4-and 5-components mixture, respectively.

Recovery factor of each metal ion depends on the composition of the mixture. The lowest recovery factors for each metal ion were obtained for 5-component mixture. In each case, the recovery factor of Zn(II) ions was the highest and amount to 90–98%.

Co(II), Zn(II), Cd(II) ions form octahedral complexes next to the tetrahedral ones. Tetrahedral complexes, being less hydrated, more readily passed to the organic phase in the extraction process (high extraction percentage), and are more easily transported through PIMs (high initial fluxes). This phenomenon creates favorable conditions for the separation of these metals in aqueous solutions.

## Figures and Tables

**Table 1:** The *pK_a_* values of EDAB-acac with other acac derivatives.

Ligand	*pK_a_*	Ref.
EDAB-acac	10.67	[This work]
pentane-2-dione (acac)	8.79	[48]
3-butyl-acetylacetone	9.60	[40]
3-allyl- acetylacetone	9.58	[40]

**Table 2:** Comparison of the stability constants *β_n_* of M(II) complexes with EDBA-acac, acac, and acac-alkyl derivatives and at 25 °C.

Ligand.	Metal ion	*log β*_1_	*log β*_2_	*log P*_1_	*log P*_2_	Ref.
acetylacetone	Cu(II)	8.01	9.20	3.15	5.40	[40]
	Co(II)	5.40	9.57			[48]
	Ni(II)	5.96	10.54			[48]
	Cu(II)	8.25	15.05			[48]
	Zn(II)	5.03	8.80			[48]
	Cd(II)	3.83	6.60			[48]
3-butyl-acetylacetonec	Cu(II)	7.95	9.45	2.78	6.19	[40]
3-allyl-acetylacetone	Cu(II)	8.32	10.26	2.96	7.38	[40]
EDAB-acac	Cu(II)	4.98	10.61	3.08	6.49	[this work]
	Co(II)	5.26	12.73	4.03	6.82	[this work]
	Ni(II)	2.15	8.85	1.44	4.71	[this work]
	Zn(II)	8.29	16.84	5.76	8.67	[this work]
	Cd(II)	5.15	14.50	4.95	7.12	[this work]

**Table 3:** Initial fluxes, order and separation coefficients for competitive transport of Co(II), Ni(II), Cu(II), Zn(II), and Cd(II) through membrane doped with EDAB-acac. Conditions as in Figure 5, Figure 6 and Figure 7.

Solutions	Metal Ions	Initial Flux, *J*_i_ µmol/m^2^∙s	Selectivity Order and Selectivity Coefficients
I	Zn(II)	10.98	-
II	Zn(II) Cd(II) Co(II)	10.53 7.48 5.39	Zn(II) > Cd(II) > Co(II) 1.4 2.0
III	Zn(II) Cu(II) Ni(II)	10.42 6.37 0.50	Zn(II) > Cu(II) >Ni(II) 1.6 21.0
IV	Zn(II) Co(II) Cu(II)	9.87 5.61 3.90	Zn(II) > Co(II) > Cu(II) 1.8 2.5
V	Zn(II) Co(II) Ni(II)	10.17 5.53 0.40	Zn(II) > Co(II) > Ni(II) 1.8 25.0
VI	Zn(II) Cd(II) Co(II) Ni(II)	9.85 7.84 5.72 0.38	Zn(II) > Cd(II) > Co(II) > Ni(II) 1.3 1.7 26.0
VII	Zn(II) Co(II) Cu(II) Ni(II)	9.94 5.89 3.61 0.40	Zn(II) > Co(II) > Cu(II) > Ni(II) 1.7 2.8 25.0
VIII	Zn(II) Cd(II) Co(II) Cu(II) Ni(II)	9.87 7.43 5.26 3.12 0.20	Zn(II) > Cd(II) > Co(II) > Cu(II) > Ni(II) 1.3 1.8 3.2 49.0

**Table 4:** Recovery factor for Zn(II), Cd(II), Co(II), Cu(II), and Ni(II) ions from ammonia solutions (pH = 7.8) after 12-h transport across PIMs with EDAB-acac

Mixture		RF, %				
		Zn	Cd	Co	Cu	Ni
I	Zn	99				
II	Zn-Cd-Co	97	82	71		
III	Zn-Cu-Ni	98			64	15
IV	Zn-Co-Cu	97		79	61	
V	Zn-Co-Ni	97		76		11
VI	Zn-Cd-Co-Ni	93	83	75		10
VII	Zn-Co-Cu-Ni	95		73	66	9
VIII	Zn-Cd-Co-Cu-Ni	90	76	64	51	5

## References

[1] Van Loon G.W., Duffy S.J. (2005). Environmental Chemistry—In a Global Perspective.

[2] Wang L.K., Chen Y.-T., Hung N., Shammas K. (2009). Heavy Metals in the Environment.

[3] Sorme L., Lagerkvist R. (2002). Sources of heavy metals in urban wastewater in Stockholm. Sci. Total Environ..

[4] Barakat M.A. (2011). New trends in removing heavy metals from industrial wastewater. Arab. J. Chem..

[5] Fu F., Wang Q. (2011). Removal of heavy metal ions from wastewaters: A review. J. Environ. Manag..

[6] Szyczewski P., Siepak J., Niedzielski P., Sobczynski T. (2009). Research on heavy metals in Poland. Pol. J. Environ. Stud..

[7] Wuana R.A., Okieimen F.E. (2011). Heavy metals in contaminated soils: A review of sources, chemistry, risks and best available strategies for remediation. Int. Sch. Res. Netw..

[8] Radzyminska-Lenarcik E., Witt K. (2020). The application of acetylacetone for the separation of heavy metals in roadside soil belts by extraction methods. Desalin. Water Treat..

[9] Kentish S.E., Stevens G.W. (2001). Innovations in separations technology for the recycling and re-use of liquid waste streams. Chem. Eng. J..

[10] Silva J.E., Paiva A.P., Soares D., Labrincha A., Castro F. (2005). Solvent extraction applied to the recovery of heavy metals from galvanic sludge. J. Hazard. Mat..

[11] Kislik V.S. (2011). Solvent Extraction: Classical and Novel Approaches.

[12] Zhang J., Hu B., Ramaswamy S., Huang H.-J., Ramarao B.V. (2013). Liquid-Liquid Extraction (LLE). Separation and Purification Technologies in Biorefineries.

[13] Regel-Rosocka M., Alguacil F.J. (2013). Recent trends in metal extraction. Rev. Metal..

[14] Witt K., Radzyminska-Lenarcik E. (2018). The recovery and the separation of metal ions from galvanic wastewaters. Desalin. Water Treat..

[15] Radzymińska-Lenarcik E., Ulewicz R., Ulewicz M. (2018). Zinc recovery from model and waste solutions using polymer inclusion membranes (PIMs) with 1-octyl-4-methylimidazole. Desalin. Water Treat..

[16] Witt K., Radzyminska-Lenarcik E. (2020). Study on effectiveness of PVC/ß-diketone sorbent in removing residue of Zn(II), Cr(III) and Ni(II) from post-galvanic wastewater. Desalin. Water Treat..

[17] Elhalawany N., Baseer R.A., Mostafa A.B., Rabei A.G. (2017). New efficient chelating polymers based on plastic waste for removal of toxic heavy metal pollutants. J. Elastom. Plast..

[18] Cote G. (2000). Hydrometallurgy of strategic metals. Solv. Extr. Ion Exch..

[19] Kołtuniewicz A.B., Drioli E. (2008). Membranes in Clean Technologies.

[20] Kislik V.S. (2010). Liquid Membranes: Principles and Applications in Chemical Separations and Wastewater Treatment.

[21] Way J.D., Noble R.D. (1992). Facilitated Transport in: Membrane Handbook.

[22] Zawierucha I., Kozłowski C.A., Malina G. (2013). Removal of toxic metal ions from landfill leachate by complementary sorption and transport across polymer inclusion membranes. Waste Manag..

[23] Costache L.N., Szczepanski P., Olteanu C., Lica C.G., Teodorescu S., Orbeci C. (2014). Bulk liquid membrane separation of different cations using D2EHPA and Cyanex 302 as carriers. Rev. Chim..

[24] De Gyves J., de San Miguel E.R. (1999). Metal ion separations by supported liquid membranes. Ind. Eng. Chem. Res..

[25] Almeida M.I.G.S., Cattrall R.W., Kolev S.D. (2012). Recent trends in extraction and transport of metal ions using polymer inclusion membranes (PIMs). J. Membr. Sci..

[26] Zulkefeli N.S.W., Weng S.K., Halim N.S.A. (2018). Removal of Heavy Metals by Polymer Inclusion Membranes. Curr. Pollut. Rep..

[27] Kolev S.D., Almeida M.I.G.S., Cattrall R.W., de la Guardia M., Esteve-Turrillas F.A. (2019). Polymer Inclusion Membranes, Smart Materials for Sensing and Separation. Handbook of Smart Materials in Analytical Chemistry.

[28] Hosseini S.S., Bringas E., Tan N.R., Ortiz I., Ghahramani M., Shahmirzadi M.A.A. (2016). Recent progress in development of high performance polymeric membranes and materials for metal plating wastewater treatment: A review. J. Water Proc. Eng..

[29] Drioli E., Romano M. (2001). Progress and new perspectives on integrated membrane operations for sustainable industrial growth. Ind. Eng. Chem. Res..

[30] Agreda D.D., Garcia-Diaz I., López F.A., Alguacil F.J. (2011). Supported liquid membranes technologies in metals removal from liquid effluents. Rev. Metal..

[31] Rynkowska E., Fatyeyeva K., Kujawski W. (2018). Application of polymer-based membranes containing ionic liquids in membrane separation processes: A critical review. Rev. Chem. Eng..

[32] Almeida M.I.G.S., Cattrall R.W., Kolev S.D. (2017). Polymer inclusion membranes (PIMs) in chemical analysis—A review. Anal. Chim. Acta.

[33] Staniszewski B., Urbaniak W. (2009). A simple and efficient synthesis of 3-substituted derivatives of pentane-2,4-dione. Chem. Pap..

[34] Alguacil F.J., Cobo A. (1999). Solvent extraction with LIX 973N for selective separation of copper and nickel. J. Chem. Technol. Biotechnol..

[35] Ochromowicz K., Jeziorek M., Wejman K. (2014). Copper(II) extraction from ammonia leach solution. Physicochem. Probl. Miner. Process..

[36] De San Miguel E.R., Hernández-Andaluz A.M., Bañuelos J.G., Saniger J.M., Aguilar J.C., de Gyves J. (2006). LIX^®^-loaded polymer inclusion membrane for copper(II) transport: 1. Composition–performance relationships through membrane characterization and solubility diagrams. Mater. Sci. Eng. A.

[37] De Gyves J., Hernández-Andaluz A.M., de San Miguel E.R. (2006). LIX^®^-loaded polymer inclusion membrane for copper (II) transport: 2. Optimization of the efficiency factors (permeability, selectivity, and stability) for LIX^®^ 84-I. J. Membr. Sci..

[38] Dziwinski E.J., Szymanowski J. (1996). Composition of copper extractant LIX 54-100. Solv. Extr. Ion Exch..

[39] Mickler W., Reich A., Uhlemann E., Bart H.J. (1996). Liquid membrane permeation of zinc, cadmium and nickel with 4-acyl-5-pyrazolones and β-diketones. J. Membr. Sci..

[40] Radzyminska-Lenarcik E., Witt K. (2018). Solvent extraction of copper ions by 3-substituted derivatives of β-diketones. Sep. Sci. Technol..

[41] Witt K., Radzyminska-Lenarcik E., Urbaniak W. (2016). Selective transport of zinc ions through a novel polymer inclusion membranes (PIMs) containing β-diketone derivatives as a carrier reagents. Sep. Sci. Technol..

[42] Radzymińska-Lenarcik E., Witt K., Bożejewiecz D. (2018). Selective transport of copper(II) ions across polymer inclusion membrane with aromatic ß-diketones as carriers. Physicochem. Probl. Miner. Process..

[43] Radzyminska-Lenarcik E., Pyszka I., Ulewicz M. (2020). Separation of Zn(II), Cr(III), and Ni(II) ions using the polymer inclusion membranes containing acetylacetone derivative as the carrier. Membranes.

[44] Takeuchi T., Böttcher A., Quezada C.M., Meade T.J., Gray H.B. (1999). Inhibition of thermolysin and human -thrombin by cobalt(III) Schiff base complexes. Bioorganic Med. Chem..

[45] Ulewicz M., Radzyminska-Lenarcik E. (2020). Application of Hydrophobic Alkylimidazoles in the Separation of Non-Ferrous Metal Ions across Plasticised Membranes—A Review. Membranes.

[46] Radzyminska-Lenarcik E., Ulewicz M. (2019). The Application of Polymer Inclusion Membranes Based on CTA with 1-alkylimidazole for the Separation of Zinc(II) and Manganese(II) Ions from Aqueous Solutions. Polymers.

[47] Braibanti A., Ostacoli G., Paoletti P., Pettit L.D., Sammartano S. (1987). Potentiometric apparatus and technique for the pH-metric measurement of metal-complex equilibrium constants. Pure Appl. Chem..

[48] Stary J., Liljenzin J.O. (1982). Critical evaluation of equilibrium constants involving acetylacetone and its metal chelates. Pure Appl. Chem..

[49] Radzyminska-Lenarcik E. (2006). The influence of the alkyl chain length on extraction equilibrium of Cu(II) complexes with 1-alkylimidazole in aqueous solution/organic solvent system. Solv. Extr. Ion Exch..

[50] Nicholls D. (1974). Complexes and First-Row Transition Elements.

[51] Rzepka M., Kulig J., Lenarcik B. (1992). Complexes of some transition cations with 7-methylpyrido [2,3-d]imidazole and 2-(2′-pyridyl)imidazole in aqueous solution. Gazz. Chim. Ital..

[52] Kurzak B., Kamecka A., Kurzak K., Jezierska J., Kafarski P. (1998). Potentiometric and spectroscopic studies of the copper(II) complexes with some aminodiphosphonic acids in aqueous solution. Polyhedron.

[53] Lenarcik B., Ojczenasz P. (2004). Investigation of the Stability Constants of Co(II) Complexes with a Homologous Series of 1-Alkylimidazoles in Aqueous Solution by Using a Partition Method with Several Solvents. Sep. Sci. Technol..

[54] Anagnostopoulos A., Hadjispyrou S. (1984). The influence of solvent polarity on the tetrahedral-octahedral equilibrium of Co(II)complexes with 3,5-dimethylpyrazole. Polyhedron.

[55] Aizawa S., Funahashi S. (2002). Octahedral−Tetrahedral Equilibrium and Solvent Exchange of Cobalt(II) Ions in Primary Alkylamines. Inorg. Chem..

[56] Radzyminska-Lenarcik E. (2007). Effect of alkyl chain length on the extraction of Cu(II) complexes with 1-alkyl-2-methylimidazole. Sep. Sci. Technol..

[57] Lenarcik B., Ojczenasz P., Kopkowski A. (2006). The Influence of the Alkyl Chain Length and Steric Effect on Stability Constants and Extractability of Co(II) Complexes with 1-Alkyl-4(5)-methylimidazoles. Sep. Sci. Technol..

[58] Lenarcik B., Rauckyte T. (2004). The Influence of Alkyl Length on Extraction Equilibria of Ni(II) Complexes with 1-Alkylimidazoles in Aqueous Solution/Organic Solvent Systems. Sep. Sci. Technol..

[59] Lenarcik B., Kierzkowska A. (2006). The Influence of Alkyl Chain Length and Steric Effect on Extraction of Zinc(II) Complexes with 1-Alkyl-2-methylimidazoles. Solv. Extr. Ion Exch..

[60] Lenarcik B., Kierzkowska A. (2004). The Influence of Alkyl Length on Stability Constants of Zn(II) Complexes with 1-Alkylimidazoles in Aqueous Solutions and Their Partition Between Aqueous Phase and Organic Solvent. Solv. Extr. Ion Exch..

[61] Radzyminska-Lenarcik E., Pyszka I., Urbaniak W. (2020). Cadmium(II) and lead(II) extraction and transport through polymer inclusion membranes with 1-alkylimidazole. Desalin. Water Treat..

[62] Rydberg J., Musakis C., Choppin G.R. (1992). Principles and Practices of Solvent Extraction.

[63] Rossotti F.J.C., Rossotti H. (1961). The Determination of Stability Constants.

[64] Gâzo J., Bersuker I.B., Garaj J., Kabešová M., Kohout J., Langfelderowá H., Melník M., Serator M., Valach F. (1976). Plasticity of the coordination sphere of Copper(II) complexes, its manifestation and causes. Coord. Chem. Rev..

[65] Danesi P.R. (1985). Separation of metal species by supported liquid membranes. Sep. Sci. Technol..

